# Effect of processing technology on chemical, sensory, and consumers' hedonic rating of seven olive oil varieties

**DOI:** 10.1002/fsn3.2717

**Published:** 2022-01-18

**Authors:** Kaouther Ben‐Hassine, Amani Taamalli, Leila Rezig, Islem Yangui, Cinzia Benincasa, Dhafer Malouche, Naziha Kamoun, Wissem Mnif

**Affiliations:** ^1^ Research Laboratory of Agricultural Production Systems and Sustainable Development High School of Agriculture Mograne Mograne Tunisia; ^2^ Department of Chemistry College of Sciences University of Hafr Al Batin Hafr Al Batin Saudi Arabia; ^3^ University of Carthage Higher School of Food Industries of Tunis Tunis Tunisia; ^4^ Laboratory of Nanobiotechnology and Valorization of Medicinal Phytoresources University of Carthage National Institute of Applied Science and Technology UR17ES22 Tunis Cedex Tunisia; ^5^ Agricultural Research Council of Italy Olive Growing and Olive Oil Industry Research Centre Rende Italy; ^6^ Engineering School of Statistics and Information Analysis University of Carthage Tunis Tunisia; ^7^ Institut de l’olivier Unité Technologie et Qualité Sfax Tunisia; ^8^ Department of Chemistry Faculty of Sciences and Arts in Balgarn University of Bisha Bisha, Saudi Arabia; ^9^ University of Manouba ISBST BVBGR‐LR11ES31 Biotechnopole Sidi Thabet Ariana Tunisia

**Keywords:** olive oil production, preference mapping, sensorial profile, virgin olive oil

## Abstract

This study established physicochemical and sensory characteristics of virgin olive oils (VOOs) and linked them to consumers’ liking using external preference mapping. We used five Tunisian and two foreign VOO varieties produced by two processing systems: discontinuous (sp) and continuous three‐phase decanter (3p). The samples were analyzed and evaluated by a panel of 274 consumers. The external preference mapping revealed five VOO clusters with a consumer preference scores rating from 40% to 65%. Consumers highly appreciated the foreign Coratina cultivar's olive oil; the main drivers being richness in polyphenols (markers of bitterness and pungency), mainly the oleuropein aglycone, and volatile compounds (markers of green fruity, green leaves, green apple, cut grassy almond, and bitterness), particularly the *trans*‐2‐hexenol. The Tunisian Chemlali (3p) oil was second highly preferred (scoring 55%). The positive drivers for olive oil preference (a profile of almond fruity green and low bitterness and pungency) are the richness in hexanal compounds. Arbequina (sp and 3p) and Chemlali (sp) were the least appreciated due to the fact that Arbequina VOO is not in the tradition of Tunisian consumers, whereas Chemchali VOO is a minor variety representing only 2% of olive oil production in Tunisia and consumed mostly in blends. The differentiation between the two processing systems depends on the variety of cultivar; consumers are able to identify the two processing system in the case of Chetoui, Leguim, and Chemchali.

## Graphical abstract

This study was conducted to establish physicochemical and sensorial profiles of five Tunisian and two foreign monovarietal virgin olive oils (VOOs) produced by two processing systems: discontinuous (sp) and continuous three‐phase decanter (3p) and to link these characteristics to consumer liking using external preference mapping.

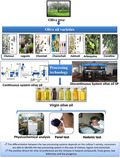

## 1 INTRODUCTION

During the last 6 years, Tunisia has contributed to 7.5% of the total world olive oil production with an average of 224.16 thousand tons and has been ranked second after the European Union (IOC, 2020). Extra‐virgin olive oil (EVOO) is a staple for most Mediterranean countries as it is a potential source of bioactive compounds, mainly tocopherols (De Bruno et al., 2020). This olive fruit juice is obtained using only mechanical processes. Its flavor and nutritional and healthy properties make it unique among other edible fats and oils, especially for its efficacy toward cardiovascular diseases, inflammation, and cancer (Francisco et al., 2019; Gouvinhas et al., 2017). EVOO is obtained using only mechanical processes that play a key role in the final VOO quality (Difonzo et al., 2021). Several other factors may affect olive oil quality such as cultivar, climate, soil quality, farming practices (traditional versus. modern methods), storage conditions (Dıraman & Dibeklioğlu, 2009; Kesen et al., 2014), genotype, and location conditions (Serrano et al., 2021).

The discontinuous process or press process was almost the only olive oil extraction system used for centuries (Abou‐Zaid, 2021). In this system, the use of water is minimal, reducing the washing off of the polyphenols and the exposition of olive paste to oxygen and light (Clodoveo et al., 2014). Over the years, the extraction process has been revolutionized to adjust to new industrial changes in the leading producer countries (Bouknana et al., 2021; Cerretani et al., 2005). Key improvement has started in the early 1970s with the invention of the centrifugation system (Vaz‐Freire et al., 2008). Currently, the extraction of olive oil using continuously a mechanical system is becoming commonly used; namely the three‐phase and the two‐phase centrifugation systems (Boudebouz et al., 2021; De Bruno et al., 2020).

Although extraction processing is a crucial factor influencing the chemical composition and the sensorial characteristics of olive oils (El‐Riachy et al., 2018), we find only few studies that have investigated the impact of extraction system on the sensory properties of VOO. Since sensory quality plays an important role in consumers’ preference, many attempts have been made to clarify the relationships between the sensory attributes in a VOO as perceived by assessors and its volatile and phenol profiles which are responsible for aroma and taste, respectively (Preedy & Watson, 2010).

To be more competitive in international markets, the olive oil industry in Tunisia is tempted to develop a business strategy based on consumers' preferences and orientations. Thus, an essential step consists in identifying the preferences of olive oil consumers and proposing a series of business strategies. The olive oil industry is subject to the advent and impacts of globalization, new commercial structures, technological advances, and consumer demands. Facing stiff competition, firms doing business in international markets need permanent improvement and innovation according to consumer preferences. A key factor for achieving this goal would be to understand consumer behavior and to identify consumer needs and desires (Parras‐Rosa et al., 2013).

During the purchase process, consumers establish preferences combining price, quality, country of origin (Dekhili et al., 2011; Mesquita & Andrade, 2014), taste, color, certification, and production method (Chrysochou et al., 2022). Zamuz et al. (2020) studied the effect of sensory and nonsensory factors on purchase intent and consumer choice. In this context, Delgado and Guinard (2011) reported disconnections on consumer behavior of olive oil between experts and ordinary consumers.

Olive oil experts use internal and external quality mapping as an efficient validation tool for the assessment of sensory quality. Such mapping uncovers potential segmentation among the experts and identifies the sensory drivers of their understanding of sensory quality. Understanding the interpretation of extra‐virgin olive oil quality is beneficial for producers and traders to commercialize olive oils. It also helps providing a clear methodology for the evaluation and the understanding of consumer preferences and expectancies for virgin olive oil.

Building on authors previous research on consumer preferences (Ben‐Hassine et al., 2014), this study investigates the main drivers of consumers' liking and disliking for selected Tunisian and foreign olive oils using external preference mapping techniques. In particular, we test whether consumers are able to differentiate between different VOO cultivars and processing systems (sp and 3p).

## 2 MATERIALS AND METHODS

### 2.1 Olive fruit sampling and processing

Olive samples of seven cultivars (Chemlali [CL], Chetoui [CT], Chemchali [CC], Leguim [L], Zalmati [Z], Arbequina [A], and Coratina [CO]) were collected in the crop year 2017–2018. For each cultivar, 10 kg of healthy olive fruits were handpicked randomly from each marked tree. One olive orchard per cultivar was used for this study and samples were taken from each marked tree to determine the ripening index before harvest. Olive oil cultivars were collected at the same ripeness index (RI) by evaluating olive skin and pulp colors (RI=3.5) according to (Uceda et al.^1998^). After collecting olives, they were mixed carefully and the splitting procedure was accomplished by submitting, for each cultivar, the same olive weight to both continuous processing systems (press system [sp]) and discontinuous one (centrifugation system with three‐phase decanter [3ph]). Oil extraction was carried out at the Olive Institute of Sfax as described in our previous work (Ben Hassine et al., 2014).

A total of 14 oil samples were obtained and directly stored in dark glass bottles for further physicochemical and sensorial analyses.

### 2.2 Panel test

The COI panel test provided by the International Olive Oil Council (IOC) was carried out for the sensory evaluation of olive oils using specific vocabulary and a standard profile sheet including positive and negative sensory attributes as described by Bendini et al. (2012). A set of three positive (fruity, pungent, and bitter) and five negative (fusty, musty, winy‐vinegar, frozen, and rancid) sensory attributes were evaluated according to the IOC norm COI/T.20/Doc. No. 15/Rev.10. The olive oil samples were evaluated for the duration of 3 hr per session with 15–20 min per sample. Samples were presented in an appropriate olive oil tasting glass according to the Glass for Tasting Oils IOC: COI/T.20/Doc. No. 5/Rev. 2/2020. The olive oil samples were presented to consumers in function of their bitterness and pungency.

The analysis was performed using a fully trained analytical taste panel composed of 11 judges (1 woman as head of the panel and 10 men, age range 28–55 years, mean age 45 years). The panelists from the Cap Bon Panel‐Tunisia (Dec‐13/103‐V/2015) recognized by the International Olive Council (IOC) since 2014 were trained according to the IOC for 1 year with continued evaluation each year according to the IOC norms (COI/T.20/Doc. No. 14/Rev. 6 2020).

### 2.3 Consumer test

Two hundred and seventy‐four consumers were recruited among trainees at the government cooker training center in Tunis‐Tunisia. They were selected according to their consumption frequency and familiarity with EVOOs. Tests were carried out on the total of 14 virgin olive oil (VOO) samples. Each sample was tasted 3 times during eight sessions (30 min per session) organized on 4 weeks in a training center specialized in food agriculture “Brevet Superior Technician.” The test was carried out in a specific room under controlled conditions to reduce external influences. The samples were presented at room temperature and served in plastic glasses coded with three‐digit numbers. A volume of 20 ml of each sample was served to taste with no obligation to finish the glass. After each test, the order of presentation of the samples was put at random. During the test, each participant evaluated the 14 samples with a 15‐min break taken after every 5 samples in order to ovoid fatigue and rinses his mouth using water or a piece of apple between each pair of VOO tastings. For each product, consumers had to rate their hedonic judgment using the labels “I like the oil” and the other “I don't like the oil,” hedonic ratings were then translated into scores ranging from 0 to 9 (Ben‐Hassine et al., 2014). The sensory trial was approved by the ethics committee of the National Institute of Nutrition and Food Technology in Tunis‐Tunisia.

### 2.4 Chemical parameters determination

#### 2.4.1 Quality indices

Free fatty acid content, peroxide value, and UV absorption characteristics were determined on the basis of the IOC method COI/T.20/Doc. No. 19/Rev. 3. The UV values were measured at 232 and 270 nm by using UV Spectrophotometer (Agilent 8453, USA).

#### 2.4.2 Oxidative stability

OSI (h) was determined using a Rancimat apparatus (Model 892 Professional Rancimat, Metrohm SA, Herisau, Switzerland) according to the method described by Tura et al. (2007). This method consists of increasing the oxidation reactions by keeping 3 g of oil at 120°C under a constant air flow of 20 L/hand, then determining the conductivity variation of water (60 ml) due to the increase in oxidative compounds.

#### 2.4.3 Pigment content

The total chlorophyll and carotenoid compounds (mg/kg) were determined calorimetrically as described by Minguez‐Mosquera et al. (1991). Olive oil samples were put into quartz cuvette and absorbance values were taken at 630, 670, and 710 nm against carbon tetrachloride for chlorophyll fraction and at 470 nm for carotenoid fraction.

#### 2.4.4 Fatty acid composition

The composition of fatty acids was evaluated as the methyl esters of fatty acids (FAME) using a cold saponification according to the method described by IOC (2018). The FAME were prepared by vigorous shaking of an oil solution in hexane (0.1 g in 2 ml) with 0.2 ml of 2 N methanolic potassium hydroxide (KOH) solution and analyzed by GC with a Hewlett‐Packard (HP 5890) chromatograph equipped with an FID detector. A fused silica column, HP‐Innowax (30 m × 0.25 mm, i.d. 0.25 µm), was used. Nitrogen was employed as a carrier gas, with a flow rate of 1 ml/min. The temperatures of the injector and detector were set at 250 and 270°C respectively. An injection volume of 1 µl was used. The operating conditions were as follows: oven temperature was held at 180°C for 1 min and then increased by 10°C/min to 220°C, held for 1 min at 220°C, increased again to 240°C at 2°C/min, and finally isotherm at 240°C for 1 min. Results were expressed as percent of relative area.

#### 2.4.5 Triacylglycerol composition

Triacylglycerols of olive oils were separated by high‐performance liquid chromatography (HPLC) equipped with a reverse phase C18 column (5 mm, 4.60 × 250 mm; Waters Associates). The eluent was monitored by refractive index detector. The mobile phase was acetone:acetonitrile (60:40 v/v) with a flow rate of 1.50 ml/min. All solvents were of HPLC grade. Samples (5 µl) were prepared by dissolving the oil in acetone (9:91 v/v). Peak assignment was carried out by comparison with chromatograms and with the retention times of some pure standards (Ben Hassine et al., 2014).

#### 2.4.6 Phenolic composition

The polar fraction was extracted by placing 5 g of oil into a 50‐ml tube containing 2 ml of hexane, and subsequently, 5 ml of methanol (water 80:20 v/v) were added. The solution was vortexed for 10 min. The emulsion was subjected to centrifugation for 20 min at 5,500*g* at 4°C to separate the two phases. The alcoholic extract was recovered, and this procedure was repeated three times. Finally, the alcoholic extract was evaporated in cold and reduced pressure conditions and the dried extract was resuspended in 1 ml of 80% methanol as described by (Montedoro et al. 1993).

The total phenolic content was determined using the spectrophotometric Folin–Ciocalteu method (Singleton & Rossi, 1965). Results are expressed as mg of hydroxytyrosol/kg of oil. The phenolic identification was performed using the Agilent 1200 Liquid Chromatography System (Agilent Technologies equipped with a standard autosampler and Agilent column Zorbax extended C_18_ 50 × 2.1 mm, 1.8 μ. The separation was carried out at 30°C with a gradient elution program at a flow rate of 0.4 ml/min. The mobile phases consisted of water plus 0.1% formic acid (A) and acetonitrile (B). The following multistep linear gradient was applied: 0 min, 10% B; 10 min, 25% B; 14 min, 50% B; 20 min, 80% B; 20 min 90% B. The injection volume in the HPLC system was 5 μl. The HPLC system was coupled to an Agilent diode array detector (DAD) (λ detection was 280 and 330 nm) and Agilent 6320 time‐of‐flight (TOF) mass spectrometer equipped with a dual electrospray interface (ESI) (Agilent Technologies) operating in negative ion mode. Detection was carried out within a mass range of 50–1700 m/*z*. Accurate mass measurements of each peak from the total ion chromatograms (TICs) were obtained by means of an Isocratic Pump (Agilent G1310B, company) using a dual nebulizer ESI source that introduces a low flow (20 μl/min) of a calibration solution that contains the internal reference masses at *m/z* 112.9856, 301.9981, 601.9790, and 1033.9881 in negative ion mode. The accurate mass data of the molecular ions were processed through the software Mass Hunter (Agilent Technologies). The quantification of phenolic compounds was achieved using calibration curves of authentic chemical standards: hydroxytyrosol, oleuropein, pinoresinol, luteolin, and apigenin.

#### 2.4.7 Volatile compounds analysis

The headspace of 2 ml of olive oil containing into a 5‐ml vial was sampled and allowed to equilibrate for 30 min using Supelco SPME devices coated with polydimethylsiloxane (PDMS, 100 µm). After the equilibration time, the fiber was exposed to the headspace for 50 min at room temperature. The fiber was then withdrawn into the needle and subjected to GC‐MS analysis. GC‐EI/MS analyses were performed with a Varian (Palo Alto, CA) CP 3800 gas chromatograph equipped with a DB‐5 capillary column (30 m × 0.25 mm, 0.25 µm; Agilent, Santa Clara, CA) and a Varian Saturn 2000 ion trap mass detector. Analytical conditions were as follows: injector and transfer line temperatures were 250 and 240°C, respectively; oven temperature was programmed from 60°C to 240°C at 3°C/min; helium was used as carrier gas at a flow rate of 1 ml/min. The identification of the volatile compounds was based on the comparison of the retention times with those of authentic standards, comparing their linear retention indices (LRI) relative to a series of n‐hydrocarbons, and on computer matching against commercial (NIST 98 and Adams) and homemade library mass spectra, built from pure substances, components of known oils, and MS literature data (Adams 1995). Moreover, the molecular weights of all the substances identified were confirmed by GC‐CI/MS, using methanol as the ionizing gas.

### 2.5 Data analysis

In order to study the effect of the product factor on all physicochemical parameters, one‐way analysis of variance (ANOVA, Tukey's honest significant difference multiple comparison) was carried out using the package agricolae (version 1.1) in the R software (version 3.0.1), which is a set of statistical procedures for agricultural research. In a further step, ascendant hierarchical clustering (AHC) was applied to the whole set of variables using the package Clust of Var version 0.5 in the R software (version 3.0.1). The aggregation criterion was the decrease in homogeneity for the cluster being merged. The homogeneity of a cluster is the sum of the squared correlation between the variables and the center of the cluster which is the first principal component of principal component analysis (PCA) mix. PCA mix is defined for a mixture of qualitative and quantitative variables and includes ordinary PCA. On the basis of the groups clustered together in the obtained dendrogram, PCA was performed to classify the products. Then, a preference mapping was performed using SensoMineR package (version 1.17) and according to the method of Danzart et al. (2004). A response surface is computed per consumer; then according to certain threshold, preference zones are delimited and finally superimposed.

## 3 RESULTS AND DISCUSSION

### 3.1 Influence of cultivar and extraction process on quality indices, pigments, volatile and phenolic compounds, and saponifiable fraction of VOOs

#### 3.1.1 Free acidity, absorbances in the UV, and peroxide value

As shown in Table 1, all the olive oil samples exhibited quality parameters within the range allowed by the regulation EC2568/91 for the extra‐virgin olive oil category (free acidity ≤0.8%; peroxide value ≤20 meq O_2_/kg; K270 ≤0.22; K232 ≤2.5) except the variety Leguim. In fact, this oil variety obtained by press system exceeded the limits established for “extra‐virgin olive oil” in free acidity (1.42%). For this reason, it could not be labeled as “extra‐virgin” according to the European Union regulations (EEC, 2003).

**TABLE 1:** Quality indices, pigments, and sensorial profile of the studied VOOs

	Arbequina		Chemchali		Chemlali		Chetoui		Coratina		Leguim		Zalmati	
	3ph	sp	3ph	sp	3ph	sp	3ph	sp	3ph	sp	3ph	sp	3ph	sp
Free acidity (% C18:1)	0.19 ± 0.01^e^	0.47 ± 0.00^cd^	0.46 ± 0.00 ^cd^	0.39 ± 0.00^de^	0.33 ± 0.00^de^	0.19 ± 0.00^e^	0.26 ± 0.01^de^	0.71 ± 0.17^bc^	0.16 ± 0.01^e^	0.32 ± 0.01^de^	0.30 ± 0.00^de^	1.42 ± 0.01^a^	0.33 ± 0.00^de^	0.77 ± 0.00^b^
K232	1.42 ± 0.20^a^	1.39 ± 0.10^a^	1.40 ± 0.14^a^	1.54 ± 0.26^a^	1.64 ± 0.13^a^	1.51 ± 0.10^a^	1.35 ± 0.11^a^	1.53 ± 0.18^a^	1.94 ± 0.02^a^	2.04 ± 0.03^a^	1.64 ± 0.21^a^	1.67 ± 0.06^a^	1.49 ± 0.17^a^	1.68 ± 0.10^a^
K270	0.10 ± 0.00^de^	0.22 ± 0.01^a^	0.17 ± 0.00^abc^	0.13 ± 0.01 ^cd^	0.10 ± 0.00^de^	0.09 ± 0.00^de^	0.18 ± 0.00^ab^	0.20 ± 0.00^ab^	0.17 ± 0.00^abc^	0.20 ± 0.00^ab^	0.09 ± 0.01^de^	0.16 ± 0.00^bc^	0.09 ± 0.01^e^	0.10 ± 0.00^de^
PV(meqO_2_/kg)	11.43 ± 0.12^abc^	11.43 ± 0.66^abc^	12.06 ± 0.06^ab^	9.43 ± 0.16^cd^	12.10 ± 0.36^ab^	11.43 ± 0.62^abc^	12.43 ± 0.63^a^	8.66 ± 0.29^de^	7.13 ± 0.56^e^	10.20 ± 0.23^bcd^	11.83 ± 0.63^ab^	12.76 ± 0.37^a^	9.06 ± 0.06^de^	13.30 ± 0.05^a^
OS(h)	4.27 ± 0.10^hi^	2.79 ± 0.09^j^	10.37 ± 0.04^c^	7.76 ± 0.08^e^	4.78 ± 0.03^gh^	3.20 ± 0.02^j^	9.53 ± 0.07^d^	6.68 ± 0.08^f^	15.37 ± 0.24^a^	13.41 ± 0.21^b^	4.78 ± 0.14^gh^	5.08 ± 0.01^g^	4.71 ± 0.03g^h^	4.08 ± 0.08^i^
Chlorophyll (ppm)	7.15 ± 0.037^a^	5.69 ± 0.02^c^	3.86 ± 0.09^e^	3.84 ± 0.18^e^	2.45 ± 0.10^f^	6.65 ± 0.25^ab^	6.49 ± 0.08a^b^	5.61 ± 0.28^c^	4.73 ± 0.07^d^	6.42 ± 0.09^b^	4.25 ± 0.01^de^	4.11 ± 0.00^de^	2.20 ± 0.08^f^	2.87 ± 0.09^f^
β‐carotene (ppm)	3.90 ± 0.02 ^cd^	3.82 ± 0.03^cd^	2.59 ± 0.06^ef^	4.21 ± 0.28^bcd^	2.41 ± 0.17^fg^	4.62 ± 0.29^abc^	5.33 ± 0.041^a^	5.29 ± 0.039^a^	5.16 ± 0.01^a^	3.43 ± 0.33^de^	4.90 ± 0.02^ab^	5.44 ± 0.19^a^	0.69 ± 0.29^b^	1.67 ± 0.06^g^
Fruity	2.93 ± 0.06^bcde^	3.06 ± 0.06^bcd^	3.16 ± 0.27^abc^	3.33 ± 0.72^abc^	3.86 ± 0.23^abc^	1.06 ± 0.06^e^	4.96 ± 0.39^a^	1.23 ± 0.14^de^	4.43 ± 0.34^ab^	3.73 ± 0.86^abc^	2.76 ± 0.06^bcde^	1.06 ± 0.06^e^	2.76 ± 0.14^bcde^	2.40 ± 0.26^cde^
Bitter	1.50 ± 0.00 ^cd^	1.70 ± 0.10 ^cd^	2.53 ± 0.20^bc^	3.56 ± 0.29^ab^	1.80 ± 0.30 cd	1.00 ± 0.00^de^	3.83 ± 0.32^a^	0.00 ± 0.00^e^	4.40 ± 0.10^a^	3.50 ± 0.25^ab^	2.43 ± 0.23^c^	0.00 ± 0.00^e^	1.80 ± 0.30 ^cd^	2.30 ± 0.10^c^
Pungent	1.40 ± 0.10^bcde^	1.86 ± 0.13^dbcd^	2.46 ± 0.03^b^	1.86 ± 0.13^bcd^	1.16 ± 0.44^de^	0.50 ± 0.28^ef^	3.83 ± 0.16^a^	0.00 ± 0.00^f^	4.73 ± 0.08^a^	2.33 ± 0.33^bc^	2.26 ± 0.18^bc^	0.00 ± 0.00^f^	0.76 ± 0.14^ef^	1.36 ± 0.27^cde^

Note

Different letters in the same line means significant difference between samples.

Acidity values of the studied oil samples ranged from 0.16% for the Coratina oil variety obtained by the three‐phase decanter (Coratina 3ph) to 1.42% for Leguim oil one obtained by press system (Leguim sp). It is worth noting that in most cases, the free acidity of oils obtained by the sp system was higher than oil samples extracted by the 3ph decanter. Such a result is consistent with previous study conducted by Ben‐Hassine et al. (2013) on two olive varieties “Chemlali” and “Coratina” extracted by super press, dual and triple phase decanter. Besides the extraction process, it is important to mention that free acidity of oils is highly influenced by other factors mainly storage conditions and time (Ghanbari Shendi et al., 2018).

The free acidity of olive oil corresponds to the proportion of fatty acids found in the free state as a result of the lipolytic action of intrinsic or extrinsic lipases. It reflects the degree of stability of the oil and its susceptibility to rancidity (Khlil et al., 2017). As reported in literature, during the SP process, olive oil is extracted with the vegetable water (aqueous phase plus solid wastes) and they remain together until they are separated by decanting, which may favor the hydrolysis of triglycerides, resulting in an increase of free fatty acids level (Torres & Maestri, 2006a, 2006b).

K232 and K270 values indicate the presence of conjugated dienes and trienes in olive oil. These molecular species are formed under the oxidation phenomenon or to the refining process. K232 and K270 values provide information on olive oil quality and are considered as indicators of the state preservation of and also show the changes due to technological processes (Khlil et al., 2017). K232 did not show any significant variation among samples, whereas K270 varied significantly from 0.09 to 0.22. The PV corresponds to the number of active oxygen's in the organic chains of the fatty substances. Expressed in meq O_2_/kg of olive oil, it evaluates the degree of oxidation of the oil. The peroxides, initially formed during the primary oxidation phase, gradually generate secondary oxidation metabolites which are volatile or nonvolatile compounds. Concerning PV, values ranged from 7.13 meqO_2_/kg for oils from “Coratina 3ph” to 13.30 meqO_2_/kg for oils from “Zalmati sp.” Generally, low peroxide values might be attributed to good extraction process, good practices in the cultivation, and favorable storage conditions of the oil (Khdair et al., 2015). In fact, it was reported by Ghanbari Shendi et al. (2019) that peroxide value increases with increasing storage time.

#### 3.1.2 Oxidative stability

Stability to oxidation is an important property for olive oil, which is improved by synergistic interactions between the lipid composition and intrinsic antioxidants. According to Najafi et al. (2015), the oxidative stability of virgin olive oil is influenced by SFA/UFA ratio and tocopherolic compounds. It is negatively affected by their fatty acid composition and minor components such as tocopherols, phytosterols, vitamin E, phenolic compounds, enzymes, and trace metals (Ghanbari Shendi et al., 2018; Gómez‐Alonso et al., 2003). Phenolic compounds reputed for their antioxidant properties play a key role for the stabilization of unsaturated fatty acids (UFA) (Miho et al., 2020).

Our results showed that cultivar and extraction system have both significant effect on oxidative stability. Among samples, Coratina olive oils obtained by both systems “sp” and “3ph” (13.41 and 15.37h, respectively) were the most stable to oxidation (Table 1). It is also important to note that olive oils obtained by the three phase system were more stable than those obtained by press system in this study. VOO are known to be more resistant to oxidation than other edible oils, thanks to their content of natural antioxidants and lower unsaturation levels (Torres & Maestri, 2006a, 2006b). A recent study conducted by Jaber Houshia et al. (2019) has shown that the relative phenolic profile highly explained the VOO oxidative stability. Their preliminary study revealed that it is possible to predict VOO oxidative stability with a regression model based on hydroxytyrosol, aldehydic open forms of oleuropein aglycone, and linoleic acid as explanatory variables.

#### 3.1.3 Pigment content

In addition to their antioxidant capacities, pigments are responsible for the color of olive oil, which is one of the factors that influence consumers thoughts and is considered as a quality parameter. Chlorophylls are responsible for the greenish color of olive oils, whereas the yellow color is due to carotenes (Psomiadou & Tsimidou, 2001). The pigment profile of olives is mainly affected by the variety (or cultivar) (Aparicio‐Ruiz et al., 2009; Lazzerini & Domenici, 2017), the ripening degree (Criado et al., 2007; Ranalli et al., 2005), and the edaphoclimatic and agronomic conditions (Jolayemi et al., 2016). Moreover, the conditions of olive oil production mostly malaxation stage and oil extraction olive oil pigment profile further influence the final content and percentage of pigments in olive oil (Ruiz‐Domínguez et al., 2013; Vaz‐Freire et al., 2008). Chlorophyll content varied from 2.20 (Zalmati 3ph) to 7.15 ppm (Arbequina 3ph). In the studied samples, β‐carotene concentration varied significantly and ranged from 0.69 ppm (Zalmati 3ph) to 5.44 ppm (Leguim sp). The extraction system and cultivar had significant effect on chlorophyll and β‐carotene amounts for the majority of our samples. However, the extraction system did not show any significant effect neither on chlorophyll levels in Chemchali and Leguim oils nor on β‐carotene levels for Arbequina, Chetoui, and Leguim oils (Table 1).

#### 3.1.4 Fatty acid and triacylglycerol composition

Fatty acid (FA) composition of the analyzed VOOs, expressed as percentage of total fatty acids (TFA), is summarized in Table 2. For all samples, the FA amounts fall within the allowed range for extra‐virgin olive oil. Palmitic (C16:0), oleic (C18:1), and linoleic (C18:2) acids were present at high levels. Palmitoleic (C16:1), linolenic (C18:3), stearic (C18:0), and arachidic (C20:0) acids were also detected, but at smaller amounts, in all the samples. Except for C14:0 and C18:0, all the FA amounts varied significantly among samples. They seemed to be influenced by the genetic factor rather than the extraction system. Regardless of the extraction system, Coratina VOO had the highest amount of C18:1 (75% and 79%, respectively) and the lowest amount of C18:2 (9% and 7%, respectively). Kelebek et al. (2015) reported significant differences, due to extraction system for C16:1, C17:0, C17:1, C18:1, C18:2, C20:0, and C18:3. Previous works confirmed this fact (Arslan & Ok, 2019; Douzane et al., 2012). Baccouri et al. (2007) reported a significant varietal impact on oleic and linoleic acid amounts.

**TABLE 2:** Fatty acid and triacylglycerol composition of the studied VOOs

FA	Arbequina		Chemchali		Chemlali		Chetoui		Coratina		Leguim		Zalmati	
	3ph	sp	Three phase	Press	Three phase	Press	Three phase	Press	Three phase	Press	Three phase	Press	Three phase	Press
C14:0	0.01 ± 0.00^a^	0.01 ± 0.00^a^	0.00 ± 0.00^a^	0.00 ± 0.00^a^	0.01 ± 0.00^a^	0.01 ± 0.00^a^	0.01 ± 0.00^a^	0.01 ± 0.00^a^	0.01 ± 0.01^a^	0.00 ± 0.00^a^	0.01 ± 0.00^a^	0.01 ± 0.00^a^	0.00 ± 0.00^a^	0.01 ± 0.00^a^
C16:0	13.73 ± 0.16^b^	16.27 ± 0.30^a^	13.01 ± 0.57^bc^	13.67 ± 0.53^b^	16.24 ± 0.34^a^	15.78 ± 0.35^a^	10.14 ± 0.29^d^	9.39 ± 0.05^d^	9.54 ± 0.41^d^	10.76 ± 0.57^d^	11.23 ± 0.32 ^cd^	9.80 ± 0.40^d^	15.89 ± 0.16^a^	16.24 ± 0.01^a^
C16:1	1.37 ± 0.20^abcd^	1.89 ± 0.04^a^	1.07 ± 0.09^abcde^	1.18 ± 0.20^abcde^	1.02 ± 0.49^abcde^	2.04 ± 0.06^a^	0.33 ± 0.12^e^	0.39 ± 0.11^de^	0.35 ± 0.08^de^	0.61 ± 0.00^bcde^	1.21 ± 0.29^abcde^	0.52 ± 0.00^cde^	1.61 ± 0.16^ab^	1.44 ± 0.25^abc^
C17:0	0.08 ± 0.00^a^	0.09 ± 0.00^a^	0.03 ± 0.00 cd	0.03 ± 0.00 ^cd^	0.05 ± 0.00^bc^	0.03 ± 0.00 ^cd^	0.05 ± 0.00^bcd^	0.05 ± 0.00^bc^	0.04 ± 0.00^bcd^	0.07 ± 0.00^ab^	0.04 ± 0.00^bcd^	0.03 ± 0.00^d^	0.03 ± 0.00^cd^	0.03 ± 0.00 ^cd^
C17:1	0.19 ± 0.02^a^	0.18 ± 0.00^a^	0.05 ± 0.00 cd	0.05 ± 0.00 cd	0.11 ± 0.01^b^	0.08 ± 0.00^bcd^	0.07 ± 0.00^bcd^	0.08 ± 0.00^bcd^	0.05 ± 0.01 cd	0.10 ± 0.00^bc^	0.06 ± 0.00^bcd^	0.04 ± 0.00^d^	0.06 ± 0.00^bcd^	0.07 ± 0.00^bcd^
C18:0	1.48 ± 0.39^a^	1.47 ± 0.17^a^	2.71 ± 0.11^a^	2.50 ± 0.37^a^	2.43 ± 0.22^a^	2.24 ± 0.29^a^	2.15 ± 0.56^a^	2.11 ± 0.44^a^	2.35 ± 0.06^a^	2.39 ± 0.08^a^	2.44 ± 0.21^a^	2.20 ± 0.32^a^	2.00 ± 0.23^a^	1.77 ± 0.34^a^
C18:1	68.51 ± 0.59 ^cd^	65.96 ± 0.52^de^	68.73 ± 0.72 ^cd^	69.22 ± 0.35 ^cd^	60.63 ± 0.76^f^	60.82 ± 0.36^f^	70.71 ± 0.82^c^	68.52 ± 0.74 cd	79.10 ± 0.76^a^	75.35 ± 0.86^ab^	72.25 ± 0.42^bc^	75.17 ± 0.22^ab^	64.03 ± 1.28^ef^	62.99 ± 1.38^ef^
C18:2	13.06 ± 0.48 ^cd^	12.81 ± 0.41 ^cd^	12.94 ± 0.53 ^cd^	11.66 ± 0.46^d^	18.02 ± 0.68^a^	17.64 ± 0.28^a^	15.08 ± 0.31^bc^	18.07 ± 0.52^a^	7.13 ± 0.14^f^	9.18 ± 0.30^ef^	11.28 ± 0.35^de^	10.93 ± 0.43^de^	14.89 ± 0.73^bc^	16.28 ± 0.52^ab^
C18:3	0.55 ± 0.11^b^	0.56 ± 0.01^b^	0.56 ± 0.07^b^	0.70 ± 0.05^ab^	0.88 ± 0.15^ab^	0.54 ± 0.03^b^	0.61 ± 0.04^ab^	0.96 ± 0.09^a^	0.67 ± 0.05^ab^	0.75 ± 0.01^ab^	0.52 ± 0.06^b^	0.59 ± 0.04^ab^	0.57 ± 0.07^b^	0.53 ± 0.07^b^
C20:0	0.63 ± 0.04^a^	0.40 ± 0.02^ab^	0.54 ± 0.07^a^	0.55 ± 0.03^a^	0.17 ± 0.13^bc^	0.55 ± 0.02^a^	0.39 ± 0.03^ab^	0.01 ± 0.00^c^	0.37 ± 0.03^ab^	0.43 ± 0.01^ab^	0.58 ± 0.10^a^	0.34 ± 0.06^ab^	0.42 ± 0.03^ab^	0.37 ± 0.06^ab^
C20:1	0.31 ± 0.02^ab^	0.31 ± 0.02^ab^	0.28 ± 0.03^ab^	0.29 ± 0.04^ab^	0.32 ± 0.06^ab^	0.24 ± 0.03^ab^	0.36 ± 0.02^ab^	0.34 ± 0.03^ab^	0.32 ± 0.02^ab^	0.32 ± 0.01^ab^	0.31 ± 0.02^ab^	0.26 ± 0.07^ab^	0.20 ± 0.01^ab^	0.13 ± 0.05^b*^
TAG (%)														
LLL	0.25 ± 0.00^d^	0.17 ± 0.00^f^	0.19 ± 0.01^ef^	0.14 ± 0.01^f^	0.53 ± 0.01^b^	0.52 ± 0.01^b^	0.41 ± 0.01^c^	0.70 ± 0.00^a^	0.06 ± 0.01^g^	0.04 ± 0.00^g^	0.23 ± 0.00^de^	0.20 ± 0.00^def^	0.36 ± 0.00^c^	0.50 ± 0.00^b^
LnLO	0.30 ± 0.00^bc^	0.31 ± 0.00^bc^	0.29 ± 0.00^c^	0.27 ± 0.00^c^	0.45 ± 0.00^a^	0.44 ± 0.00^a^	0.35 ± 0.00^b^	0.48 ± 0.00^a^	0.15 ± 0.00^d^	0.12 ± 0.00^d^	0.30 ± 0.00^c^	0.27 ± 0.00^c^	0.46 ± 0.20^a^	0.44 ± 0.10^a^
LnLP	0.05 ± 0.00^de^	0.09 ± 0.00^cde^	0.10 ± 0.00^bcd^	0.09 ± 0.00^cde^	0.15 ± 0.00^ab^	0.13 ± 0.00^bc^	0.11 ± 0.01^bc^	0.12 ± 0.01^bc^	0.05 ± 0.01^de^	0.05 ± 0.00^de^	0.09 ± 0.01^cde^	0.05 ± 0.00^e^	0.19 ± 0.01^a^	0.20 ± 0.01^a^
LLO	4.59 ± 0.01^f^	4.14 ± 0.00^g^	3.61 ± 0.00^h^	3.02 ± 0.00^i^	6.55 ± 0.01^b^	6.52 ± 0.01^b^	5.63 ± 0.02^d^	7.83 ± 0.02^a^	1.11 ± 0.01^l^	1.35 ± 0.02^k^	3.59 ± 0.01^h^	2.62 ± 0.01^j^	5.42 ± 0.01^e^	5.83 ± 0.01^c^
LnOO	1.94 ± 0.00^ef^	2.00 ± 0.00^ef^	1.92 ± 0.00^fg^	1.82 ± 0.00^gh^	3.45 ± 0.01^c^	3.87 ± 0.00^a^	2.03 ± 0.04^e^	2.60 ± 0.01^d^	1.71 ± 0.00^ij^	1.46 ± 0.03^k^	1.77 ± 0.01^hi^	1.63 ± 0.00^j^	3.43 ± 0.00^c^	3.69 ± 0.00^b^
PLL	0.62 ± 0.00^d^	0.71 ± 0.01^c^	0.46 ± 0.00^e^	0.44 ± 0.01^e^	0.84 ± 0.01^b^	0.57 ± 0.01^d^	0.29 ± 0.01^g^	0.41 ± 0.01ef	0.45 ± 0.01^e^	0.56 ± 0.00d	0.38 ± 0.00f	0.40 ± 0.00ef	0.71 ± 0.00c	0.95 ± 0.00a
LOO	17.46 ± 0.10^de^	17.17 ± 0.04^def^	17.01 ± 0.05^ef^	15.66 ± 0.04^h^	18.72 ± 0.00^c^	18.41 ± 0.00^c^	20.40 ± 0.01^b^	21.85 ± 0.31^a^	11.77 ± 0.03^j^	12.67 ± 0.05^i^	17.53 ± 0.02^d^	16.41 ± 0.03^g^	17.56 ± 0.05^d^	16.76 ± 0.02^fg^
LOP	11.04 ± 0.00^f^	11.71 ± 0.00^e^	10.46 ± 0.01^g^	9.69 ± 0.01^h^	15.16 ± 0.00^b^	15.05 ± 0.00^c^	8.31 ± 0.02^j^	8.82 ± 0.01i	5.13 ± 0.01m	4.55 ± 0.03^n^	7.30 ± 0.01^k^	5.68 ± 0.01^l^	14.26 ± 0.00^d^	15.36 ± 0.00^a^
PLP	1.13 ± 0.00^f^	1.20 ± 0.00^e^	0.96 ± 0.00^g^	0.92 ± 0.00^h^	1.84 ± 0.00^c^	2.10 ± 0.00^b^	0.63 ± 0.00^j^	0.71 ± 0.00i	0.50 ± 0.00k	0.37 ± 0.01^l^	0.51 ± 0.00^k^	0.34 ± 0.01^l^	1.62 ± 0.00^d^	2.35 ± 0.00^a^
OOO	31.41 ± 0.34^d^	31.14 ± 0.13^d^	32.10 ± 0.13^d^	33.43 ± 0.14 ^cd^	21.98 ± 0.00^e^	21.94 ± 0.01^e^	36.07 ± 0.14^c^	33.73 ± 0.13 ^cd^	48.82 ± 0.00^a^	49.44 ± 0.18^a^	43.08 ± 1.52^b^	41.74 ± 1.18^b^	23.31 ± 0.01^e^	21.05 ± 0.00^e^
POP	22.10 ± 0.08^de^	23.21 ± 0.07^b^	23.44 ± 0.18^b^	24.23 ± 0.21^a^	21.57 ± 0.07^ef^	21.46 ± 0.07^f^	17.68 ± 0.06^i^	15.65 ± 0.09^j^	21.60 ± 0.09^ef^	21.50 ± 0.22_f_	19.90 ± 0.01^g^	19.17 ± 0.04^h^	22.87 ± 0.01^bc^	22.53 ± 0.01 ^cd^
POO	3.93 ± 0.06 ^cd^	3.49 ± 0.67^de^	3.95 ± 0.06 ^cd^	4.31 ± 0.07^bcd^	4.86 ± 0.02^abc^	4.52 ± 0.11^bc^	2.13 ± 0.06^f^	1.90 ± 0.06^f^	2.48 ± 0.02^f^	2.45 ± 0.0^4f^	2.52 ± 0.06^ef^	2.16 ± 0.06^f^	5.17 ± 0.07^ab^	5.65 ± 0.07^a^
AOL	0.46 ± 0.00 ^cd^	0.36 ± 0.00^d^	0.40 ± 0.02^d^	0.37 ± 0.02^d^	0.24 ± 0.02^e^	0.21 ± 0.02^e^	0.52 ± 0.00^bc^	0.38 ± 0.00^d^	0.86 ± 0.03^a^	0.76 ± 0.03^a^	0.47 ± 0.00 ^cd^	0.59 ± 0.00^b^	0.24 ± 0.00^e^	0.18 ± 0.00^e^
SOO	3.05 ± 0.01^e^	2.95 ± 0.01^e^	4.62 ± 0.01^b^	4.78 ± 0.01^b^	2.62 ± 0.01^e^	3.05 ± 0.01^e^	4.74 ± 0.05^b^	4.04 ± 0.05^c^	4.70 ± 0.01^b^	4.61 ± 0.08^b^	4.72 ± 0.05^b^	5.47 ± 0.05^a^	3.27 ± 0.01^d^	3.15 ± 0.01^de^
SOP	0.96 ± 0.00^efg^	0.82 ± 0.00^g^	1.24 ± 0.00^bcd^	1.45 ± 0.00^ab^	0.84 ± 0.00^fg^	1.08 ± 0.00^de^	1.06 ± 0.06^def^	0.91 ± 0.06^efg^	0.88 ± 0.00^efg^	0.76 ± 0.03^g^	0.92 ± 0.05^efg^	1.09 ± 0.06^cde^	1.30 ± 0.06^abc^	1.50 ± 0.06^a^

Note

Different letters in the same line means significant difference between samples.

It is noteworthy that high levels of unsaturated fatty acids (FAs) (mainly C18:1) and several antioxidants of olive oil have beneficial effects on human health. Due to their positive effect on serum cholesterol levels, olive oils with higher monounsaturated FA (MUFA) and lower saturated FAs are preferred (Clodoveo et al., 2014).

Regarding triacylglycerols, significant differences were noticed among the analyzed samples (Table 2). A significant effect of the extraction system on some triacylglycerols was reported in literature (Kelebek et al., 2015). Cultivar also showed a significant effect on the triacylglycerol composition of olive oil (Salvador et al., 2003).

Among the main triacylglycerols (LOO, LOP, OOO), the percentage of OOO was the highest (Table 2) ranging from the simple (21% in Chemlali and Leguim oils) to the double (49% in Coratina oil). LOP levels varied also largely from 4% (Coratina oil) to 15% (Zalmati oil). These results are in accordance with those reported in literature (Salvador et al., 2003).

Table 3 shows the variation of the phenolic composition of the analyzed samples. The total phenol content varied largely between 160.54 (Arbequina sp) and 525.18 mg/kg (Coratina sp). The cultivar effect on the phenolic content in VOO was well reported and established in literature (Kivrak et al., 2020; Visioli & Galli, 2002). Oils obtained from the cultivar Coratina had the highest amount of phenols (525.18 mg/kg). However, the phenol levels were influenced by the extraction system. For the cultivars Coratina, Leguim, Zalmati, and Chetoui, the total phenol content was higher for oils extracted by the press system in contrast to the rest of cultivars (Arbequina, Chemlali, and Chemchali). In press extraction process, the amount of added water is minimal when compared with the continuous system, thus reducing polyphenols washing off. Phenols present in olive paste are soluble in both water and oil, depending on their partition coefficients and the temperature. Addition of water to the paste alters the partition equilibrium between aqueous and oil phases and causes a reduction of phenolic concentrations through dilution of the aqueous phase. Besides, a coincidental lower concentration of these substances occurs in the oil phase. As explained by Clodoveo (2012), higher water/paste ratios are used in triple phase centrifugation, and therefore larger amounts of phenols are eliminated with water wastes. In addition, during the crushing phase, the softer action exerted by the toothed disc crusher, compared with the one exerted by the hammer crusher, should influence the enzymatic activity and, consequently, the total phenolic content. In fact, oil phenol losses during extraction steps, decreasing then its antioxidant property (Haddada Mahjoub et al., 2007).

**TABLE 3:** Phenolic composition of the studied VOOs

Phenolic compound (ppm)	Arbequina		Chemchali		Chemlali		Chetoui		Coratina		Leguim		Zalmati	
	Three phase	Press	Three phase	Press	Three phase	Press	Three phase	Press	Three phase	Press	Three phase	Press	Three phase	Press
Hydroxytyrosol	0.20 ± 0.00^de^	0.23 ± 0.00^d^	0.92 ± 0.01^b^	0.14 ± 0.00^fgh^	0.15 ± 0.00^fg^	0.13 ± 0.00^gh^	0.71 ± 0.00^c^	1.92 ± 0.00a	0.94 ± 0.00^b^	0.14 ± 0.00^fgh^	0.04 ± 0.00^i^	0.10 ± 0.00^h^	0.17 ± 0.00e^f^	0.21 ± 0.00^d^
Tyrosol	1.09 ± 0.01^e^	0.33 ± 0.00^i^	5.21 ± 0.01^a^	1.41 ± 0.00^d^	0.63 ± 0.01^g^	0.84 ± 0.01^f^	1.08 ± 0.02^e^	4.10 ± 0.02^b^	2.25 ± 0.01^c^	1.42 ± 0.00^d^	0.04 ± 0.00^k^	0.12 ± 0.00^j^	0.18 ± 0.00j	0.55 ± 0.00^h^
DFOA	10.20 ± 0.032^cde^	10.11 ± 0.07^de^	10.09 ± 0.04^e^	10.74 ± 0.03^bcd^	10.19 ± 0.22^cde^	11.41 ± 0.04^a^	10.37 ± 0.02^cde^	10.46 ± 0.07^bcde^	10.77 ± 0.16^bc^	10.16 ± 0.03^cde^	11.09 ± 0.19^ab^	10.00 ± 0.23^e^	10.58 ± 0.05^bcde^	10.00 ± 0.11^e^
DFLA	10.11 ± 0.06^d^	10.32 ± 0.05^d^	11.35 ± 0.04^c^	10.98 ± 0.07^c^	10.27 ± 0.05^d^	10.06 ± 0.08^d^	20.63 ± 0.25^b^	40.62 ± 0.05^a^	20.86 ± 0.16^b^	10.98 ± 0.07^c^	10.07 ± 0.02^d^	20.76 ± 0.26^b^	11.38 ± 0.07^c^	10.11 ± 0.09^d^
Ac‐Pin	0.18 ± 0.00^c^	0.43 ± 0.00^c^	1.55 ± 0.18^b^	1.20 ± 0.01^b^	0.10 ± 0.00^c^	0.03 ± 0.00^c^	3.27 ± 0.00^a^	1.23 ± 0.01^b^	1.30 ± 0.01^b^	1.13 ± 0.03^b^	1.22 ± 0.01^b^	0.17 ± 0.00^c^	0.12 ± 0.00c	0.42 ± 0.33^c^
Pin	8.12 ± 0.01^b^	7.78 ± 0.06^c^	1.71 ± 0.05^h^	0.12 ± 0.01^jk^	2.71 ± 0.03^f^	0.25 ± 0.01^j^	9.19 ± 0.04^a^	5.52 ± 0.01e	7.62 ± 0.01^d^	0.13 ± 0.00^jk^	1.91 ± 0.00^g^	0.09 ± 0.00^k^	1.35 ± 0.00i	0.18 ± 0.00^jk^
EA	0.76 ± 0.01^f^	0.75 ± 0.00^f^	4.36 ± 0.00^a^	3.27 ± 0.01^b^	0.63 ± 0.^01g^	0.07 ± 0.00^h^	4.40 ± 0.04^a^	2.94 ± 0.01c	1.16 ± 0.01^e^	3.25 ± 0.01^b^	0.11 ± 0.00^h^	0.08 ± 0.00^h^	2.64 ± 0.01^d^	0.74 ± 0.01^f^
OA	23.50 ± 0.05^g^	10.70 ± 0.27^h^	40.67 ± 0.10^e^	50.92 ± 0.10^c^	45.44 ± 0.08^d^	40.23 ± 0.13	50.72 ± 0.15^c^	30.70 ± 0.17^f^	30.86 ± 0.22^f^	70.92 ± 0.14^a^	10.00 ± 0.40^h^	50.69 ± 0.08^c^	51.35 ± 0.19^c^	61.35 ± 0.06^b^
LA	0.32 ± 0.02^fg^	0.40 ± 0.04^f^	4.93 ± 0.09^b^	1.72 ± 0.04^e^	0.18 ± 0.04^fgh^	0.19 ± 0.03^fgh^	5.55 ± 0.16^a^	2.26 ± 0.05^d^	2.62 ± 0.02^c^	1.72 ± 0.01^e^	0.00 ± 0.00^h^	0.14 ± 0.04^fgh^	0.22 ± 0.00f^gh^	0.04 ± 0.02^gh^
Total polyphenols	198.23 ± 0.72^gh^	160.54 ± 0.32^i^	424.06 ± 0.32^c^	294.31 ± 0.14^e^	285.60 ± 0.23^ef^	186.40 ± 0.42^h^	424.65 ± 0.51^c^	476.92 ± 10.30^b^	379.93 ± 8.47^d^	525.18 ± 1.71^a^	217.42 ± 1.91g	301.58 ± 0.20^e^	215.56 ± 0.56^g^	264.59 ± 6.74^f^

Note

Different letters in the same line means significant difference between samples.

Abbreviations: DFLA, dialdehydic form of ligstroside aglycon; DFOA, dialdehydic form of oleuropein aglycon; EA, elenolic acid; LA, ligstroside aglycon; OA, oleuropein aglycon; Pin, pinoresinol.

Nine phenolic compounds were identified in the analyzed samples: the secoiridoid oleuropein aglycon, its dialdehydic form (DFOA), and the dialdehydic form of ligstroside aglycone (DFLA) were the major compounds (Table 3). Oleuropein and ligstroside aglycones’ concentrations varied largely among samples according to the extraction process and cultivar, being the highest levels registered for “Coratina sp” and “Chetoui sp” oils, respectively (Table 3). Hydroxytyrosol concentrations varied significantly according to the extraction system where the highest ones were registered for three‐phase decanter in the case of Chemchali, Chemlali, and Coratina VOOs. The same fact was noticed for tyrosol whose concentrations were higher in Arbequina, Coratina, and Chemchali oils from three‐phase decanter processing than in those with the super press. Like in the case of hydroxytyrosol, pinoresinol concentrations showed significant variation among samples extracted with pressure and centrifugation regardless the cultivar. It is important to mention that oils produced from three‐phase processing were richer in these phenolics than those obtained by pressure. Other factors were reported to influence the phenolic compounds concentrations in olive oils such as storage time and conditions. Ghanbari Shendi et al. (2018) demonstrated that phenolic compound concentrations significantly decline after a year storage time.

Although a full description of the organoleptic characteristics of the oil is only obtainable through sensory analysis, the quali‐quantitative determination of the volatile compounds can provide very useful information on product quality (Angerosa et al., 2004).

The main volatiles contributing to the peculiar aroma VOO are C6 and C5 biogenerated through the lipoxygenase pathway during oil production (Brkić Bubola et al., 2012). The concentration and activity of enzymes involved in this biogenesis are influenced by several agronomical and technological factors, among them are cultivar (Runcio et al., 2008), stage of olive ripeness, and production conditions (Olias et al., 1993).

In our study, several compounds belonging to different chemical classes (carbonyl: aldehydes and ketones, alcohols, esters, hydrocarbons, some acids, and furane derivatives) were detected (Table 4). The chemical composition of all tested olive oils showed that C_6_ compounds (*trans*‐2‐hexenal, *cis*‐3‐hexenal, hexanol, and *cis*‐3‐hexenol) were the most abundant compounds. For the monovarietal olive oils involved in this study, *trans*‐2‐hexenal was the major C_6_ aldehyde volatile in Coratina olive oils (372.41 ppm). However, olive oils obtained from Leguim variety contained the lowest amount (1.24 ppm). The C_6_ aldehydes hexanal and *trans*‐2‐hexenal, as well as hexanol, contribute to the typical green sensory perception. Produced via the LOX pathway from polyunsaturated fatty acids (linolenic and linoleic acids), hexanal and *trans*‐2‐hexenal accumulate in virgin olive oils during physical extraction procedures (Iraqi et al., 2005). The latter is derived from *cis*‐3‐hexenal, which undergoes isomerization to a more stable compound that can then be further reduced to *trans*‐2‐hexen‐1‐ol (Luna et al., 2006). Hexyl and *trans*‐3‐hexenyl acetate were present in the aroma of all our samples, but at different levels. These esters are synthesized by alcohol acyltransferase (AAT) within the LOX pathway. Low levels of esters in “Coratina,” “Zalmati,” and “Chemlali” sp VOO samples indicate a lower content of AAT. It is also important to note the high level of 1‐hexanol in “Chemlali sp” (but detected in trace levels in Leguim and Chemchali), which emphasizes the perception of fruity and aromatic pleasant attributes (Brkić Bubola et al., 2012).

**TABLE 4:** Volatile compounds of the studied VOOs

Volatile compound (ppm)	Arbequina		Chemchali		Chemlali		Chetoui		Coratina		Leguim		Zalmati	
	Three phase	Press	Three phase	Press	Three phase	Press	Three phase	Press	Three phase	Press	Three phase	Press	Three phase	Press
Octane	0.51 ± 0.04^j^	6.27 ± 0.09^a^	3.32 ± 0.03^c^	0.49 ± 0.03^j^	4.40 ± 0.02^b^	1.79 ± 0.05^g^	2.33 ± 0.05^f^	0.71 ± 0.01^ij^	1.28 ± 0.03^h^	0.91 ± 0.04^i^	2.56 ± 0.03^e^	0.53 ± 0.03^j^	2.87 ± 0.01^d^	2.66 ± 0.03^de^
Acetone	0.22 ± 0.01^i^	3.74 ± 0.09^abc^	0.59 ± 0.04^hi^	3.83 ± 0.07^ab^	3.69 ± 0.18^bc^	4.17 ± 0.08^a^	2.23 ± 0.10^e^	2.75 ± 0.08^d^	3.32 ± 0.10^c^	1.42 ± 0.06^fg^	0.69 ± 0.05^h^	1.64 ± 0.02^f^	1.03 ± 0.04^gh^	1.54 ± 0.06^f^
Ethylacetate	2.25 ± 0.10^h^	3.30 ± 0.11^h^	3.38 ± 0.15^h^	39.01 ± 0.09^b^	98.92 ± 0.62^a^	29.07 ± 0.35^c^	14.84 ± 0.36^ef^	26.39 ± 0.61^d^	13.60 ± 0.21^f^	8.25 ± 0.16^g^	7.24 ± 0.38^g^	15.64 ± 0.29^e^	13.53 ± 0.40^f^	30.63 ± 0.31^c^
2‐Butanone	4.07 ± 0.05^fg^	0.94 ± 0.03^i^	5.70 ± 0.06^e^	0.00 ± 0.00^j^	3.93 ± 0.09^gh^	4.35 ± 0.12^fg^	3.49 ± 0.14^h^	0.20 ± 0.00^j^	13.06 ± 0.06^b^	8.55 ± 0.06^c^	7.21 ± 0.14^d^	0.25 ± 0.00^j^	21.12 ± 0.11^a^	4.48 ± 0.16^f^
2‐Methylbutanal	0.23 ± 0.01^fg^	1.52 ± 0.06^a^	0.65 ± 0.01 ^cd^	0.00 ± 0.00^g^	1.64 ± 0.02^a^	0.42 ± 0.01^def^	0.77 ± 0.02^c^	0.29 ± 0.02^ef^	1.07 ± 0.02^b^	0.00 ± 0.00^g^	0.35 ± 0.17^ef^	0.38 ± 0.01^def^	0.00 ± 0.00^g^	0.55 ± 0.02^cde^
3‐Methylbutanal	0.34 ± 0.01^g^	0.77 ± 0.05^f^	0.95 ± 0.00^e^	0.00 ± 0.00^j^	1.10 ± 0.02^d^	0.29 ± 0.00^gh^	1.99 ± 0.02^b^	0.10 ± 0.01^ij^	1.53 ± 0.00^c^	0.35 ± 0.01^g^	2.12 ± 0.03^a^	0.19 ± 0.00^hi^	0.00 ± 0.00^j^	0.86 ± 0.01^ef^
Isopropanol	5.33 ± 0.02^h^	5.63 ± 0.09^h^	7.22 ± 0.02^de^	4.47 ± 0.00^i^	8.80 ± 0.015^b^	7.49 ± 0.01 cd	10.97 ± 0.01^a^	2.11 ± 0.00^j^	5.95 ± 0.02^g^	6.55 ± 0.17^f^	11.08 ± 0.10^a^	1.57 ± 0.01^k^	7.73 ± 0.01^c^	7.09 ± 0.01^e^
2‐Ethylfurane	0.00 ± 0.00^g^	0.75 ± 0.03^d^	0.35 ± 0.01^ef^	0.45 ± 0.01^e^	5.05 ± 0.08^a^	0.87 ± 0.03^d^	0.32 ± 0.01^ef^	1.33 ± 0.05^b^	0.27 ± 0.01^f^	0.00 ± 0.00^g^	1.13 ± 0.05^c^	0.23 ± 0.00^f^	0.32 ± 0.01^ef^	0.47 ± 0.01^e^
2‐Pentanone−3‐pentanone	15.91 ± 0.10^f^	18.97 ± 0.13^e^	4.49 ± 0.15^j^	4.98 ± 0.06^j^	42.36 ± 0.08^a^	6.91 ± 0.02^hi^	26.09 ± 0.76^c^	5.71 ± 0.08^ij^	32.12 ± 0.41^b^	26.70 ± 0.12^c^	7.93 ± 0.10^h^	5.49 ± 0.08^j^	24.27 ± 0.32^d^	13.34 ± 0.16^g^
2‐Butanol	0.21 ± 0.01^g^	0.90 ± 0.02^b^	0.24 ± 0.01^g^	0.21 ± 0.00^g^	0.55 ± 0.01 cd	0.44 ± 0.01^def^	0.47 ± 0.02^de^	0.31 ± 0.02^fg^	0.94 ± 0.01^b^	0.32 ± 0.00^fg^	0.35 ± 0.02^efg^	0.39 ± 0.01^ef^	0.62 ± 0.05^c^	1.17 ± 0.06^a^
Penten−3‐one	0.26 ± 0.01^de^	0.81 ± 0.03^d^	1.61 ± 0.01^c^	0.39 ± 0.31^de^	1.63 ± 0.05^c^	0.00 ± 0.00^e^	14.20 ± 0.03^a^	0.43 ± 0.34^de^	0.82 ± 0.01^d^	0.24 ± 0.00^de^	8.70 ± 0.04^b^	0.00 ± 0.00^e^	0.00 ± 0.00^e^	0.04 ± 0.00^e^
Propanol	0.00 ± 0.00^g^	0.01 ± 0.00^g^	0.12 ± 0.00^f^	0.52 ± 0.01^b^	1.10 ± 0.01^a^	0.23 ± 0.01^d^	0.14 ± 0.00^ef^	0.18 ± 0.01^e^	0.43 ± 0.01^c^	0.00 ± 0.00^g^	0.14 ± 0.00^ef^	0.25 ± 0.01^d^	0.11 ± 0.00^f^	0.00 ± 0.00^g^
3‐Hexanone	0.71 ± 0.01^h^	5.89 ± 0.03^c^	1.43 ± 0.03^g^	5.37 ± 0.23^d^	12.62 ± 0.15^a^	6.32 ± 0.06^c^	1.69 ± 0.03^g^	3.24 ± 0.09^f^	1.42 ± 0.00^g^	1.71 ± 0.08^g^	1.41 ± 0.02^g^	3.93 ± 0.08^e^	3.22 ± 0.05^f^	9.32 ± 0.05^b^
Hexanal	9.15 ± 0.04^i^	35.41 ± 0.19^b^	22.66 ± 0.09^e^	3.70 ± 0.04^l^	34.11 ± 0.10^c^	18.20 ± 0.04^f^	15.15 ± 0.07^g^	7.68 ± 0.07^k^	9.84 ± 0.03^h^	7.32 ± 0.13^k^	28.55 ± 0.02^d^	3.15 ± 0.04^m^	8.66 ± 0.07^j^	43.34 ± 0.09^a^
Isobutanol	1.47 ± 0.03^i^	32.52 ± 0.19^c^	2.18 ± 0.01^hi^	22.61 ± 0.09^f^	76.44 ± 0.61^a^	53.42 ± 0.67^b^	4.87 ± 0.07^g^	23.30 ± 0.13^f^	3.39 ± 0.10^h^	1.90 ± 0.18^i^	2.09 ± 0.03^hi^	25.51 ± 0.07^e^	2.80 ± 0.02^hi^	28.79 ± 0.11^d^
3‐Pentanol	0.33 ± 0.00^i^	1.22 ± 0.05^e^	0.07 ± 0.00^j^	0.66 ± 0.00^fg^	2.93 ± 0.00^b^	3.24 ± 0.01^a^	1.32 ± 0.01^e^	0.54 ± 0.01^h^	2.49 ± 0.04^c11^	0.67 ± 0.017^fg^	0.22 ± 0.00^i^	0.64 ± 0.01^gh^	0.76 ± 0.00^h^	1.84 ± 0.01^d^
*Trans*−2‐pentenal	0.19 ± 0.01^ef^	0.50 ± 0.03^de^	0.22 ± 0.00^ef^	0.32 ± 0.00^def^	1.02 ± 0.02^bc^	0.00 ± 0.00^f^	1.50 ± 0.03^a^	0.04 ± 0.00^f^	1.13 ± 0.00^ab^	0.24 ± 0.01^ef^	0.94 ± 0.02^bc^	0.08 ± 0.01^ef^	0.70 ± 0.30 ^cd^	0.20 ± 0.01^ef^
Butanol	0.21 ± 0.01^h^	0.92 ± 0.03^c^	0.44 ± 0.01^f^	0.32 ± 0.00^g^	1.65 ± 0.00^a^	0.94 ± 0.00^c^	0.66 ± 0.01^e^	0.16 ± 0.00^h^	1.65 ± 0.01^a^	0.00 ± 0.00^i^	0.18 ± 0.00^h^	0.36 ± 0.00^g^	0.84 ± 0.01^d^	1.31 ± 0.01^b^
1‐Penten−3‐ol	5.89 ± 0.04^i^	13.15 ± 0.25^g^	12.84 ± 0.01^g^	4.72 ± 0.03^k^	36.94 ± 0.02^d^	6.24 ± 0.02^i^	25.79 ± 0.07^c^	3.47 ± 0.02l	22.88 ± 0.03^d^	13.73 ± 0.15^f^	31.76 ± 0.12^b^	5.28 ± 0.03^j^	22.13 ± 0.03^e^	11.53 ± 0.01^h^
*Cis*−3‐hexenal	3.16 ± 0.03^d^	23.02 ± 0.92^b^	2.09 ± 0.02^d^	22.92 ± 0.04^b^	46.01 ± 0.14^a^	38.27 ± 10.14^a^	4.49 ± 0.02 cd	0.00 ± 0.00^d^	3.23 ± 0.03^d^	3.53 ± 0.21^d^	1.91 ± 0.02^d^	19.92 ± 0.03^b^	2.83 ± 0.00^d^	17.69 ± 0.04^bc^
Isopentanol	4.17 ± 0.02^e^	42.17 ± 10.90^d^	6.34 ± 0.01^e^	75.21 ± 0.13^c^	227.49 ± 0.24^a^	116.79 ± 0.41^b^	9.86 ± 0.01^e^	105.15 ± 0.22^b^	6.72 ± 0.03^e^	5.13 ± 0.09^e^	3.52 ± 0.02^e^	42.09 ± 0.06^d^	6.37 ± 0.02^e^	45.48 ± 0.05^d^
*Trans*−2‐hexenal	73.18 ± 0.44^g^	148.60 ± 0.46^d^	16.72 ± 0.05^k^	3.57 ± 0.05l	62.28 ± 0.27^i^	24.25 ± 0.52^j^	71.04 ± 0.30^h^	3.13 ± 0.03l	372.41 ± 0.39^a^	161.18 ± 0.49^b^	158.99 ± 0.13^c^	1.24 ± 0.02^m^	80.05 ± 0.42^f^	121.59 ± 0.49^e^
Pentanol	0.21 ± 0.01^e^	0.25 ± 0.02^de^	0.11 ± 0.00^f^	0.00 ± 0.00^h^	0.41 ± 0.01^a^	0.46 ± 0.01^ab^	0.36 ± 0.01^bc^	0.24 ± 0.01^e^	0.08 ± 0.00^fg^	0.04 ± 0.00^gh^	0.11 ± 0.00^f^	0.31 ± 0.00 ^cd^	0.22 ± 0.00^e^	0.31 ± 0.00^c^
Hexylacetate	4.22 ± 0.01^g^	4.39 ± 0.23^g^	8.92 ± 0.02^e^	3.79 ± 0.04^h^	10.00 ± 0.01^b^	9.91 ± 0.02^bc^	9.89 ± 0.01^bc^	1.75 ± 0.02^i^	7.18 ± 0.02^f^	9.26 ± 0.12^de^	10.43 ± 0.01^a^	1.30 ± 0.02^j^	10.56 ± 0.01^a^	9.55 ± 0.02 ^cd^
Octanal	0.25 ± 0.00^de^	0.48 ± 0.01^c^	0.51 ± 0.00^bc^	0.22 ± 0.00^e^	0.77 ± 0.05^a^	0.53 ± 0.00^bc^	0.55 ± 0.00^bc^	0.13 ± 0.00^f^	0.33 ± 0.00^d^	0.48 ± 0.01^c^	0.54 ± 0.00^bc^	0.00 ± 0.00^g^	0.60 ± 0.00^b^	0.76 ± 0.00^a^
*Cis*−3‐hexenyl‐acetate	7.12 ± 0.02^g^	12.59 ± 0.20^f^	22.17 ± 0.01^e^	27.65 ± 0.02^c^	27.10 ± 0.04^d^	1.77 ± 0.01^j^	57.23 ± 0.05^a^	7.16 ± 0.01^g^	1.75 ± 0.00^jk^	1.81 ± 0.08^j^	29.23 ± 0.01^b^	6.17 ± 0.02^h^	2.73 ± 0.01^i^	1.43 ± 0.01^k^
*Cis*−2‐pentenol	3.42 ± 0.01^j^	8.38 ± 0.18^f^	5.77 ± 0.07^h^	3.35 ± 0.01^j^	20.19 ± 0.02^a^	5.33 ± 0.04^i^	15.34 ± 0.01^b^	2.86 ± 0.00^k^	12.32 ± 0.00^d^	8.34 ± 0.08^f^	14.92 ± 0.03^c^	2.91 ± 0.02^k^	9.74 ± 0.02^e^	6.63 ± 0.034^g^
*Trans*−2‐heptenal	0.00 ± 0.00^k^	0.52 ± 0.02^c^	0.11 ± 0.00^j^	0.00 ± 0.00^k^	1.20 ± 0.00^a^	0.44 ± 0.00^d^	0.33 ± 0.00^e^	0.35 ± 0.00^e^	0.21 ± 0.00^g^	0.15 ± 0.00^ij^	0.16 ± 0.00^hi^	0.28 ± 0.00^f^	0.19 ± 0.00^gh^	0.62 ± 0.00^b^
6‐Methyl−5‐hepten−2‐one	0.17 ± 0.00^hij^	0.18 ± 0.01^hi^	0.36 ± 0.00^e^	0.15 ± 0.00^ij^	1.55 ± 0.01^a^	0.34 ± 0.00^ef^	0.35 ± 0.00^e^	0.51 ± 0.00^d^	0.12 ± 0.00^j^	0.28 ± 0.02^g^	0.90 ± 0.01^c^	0.21 ± 0.00^h^	1.30 ± 0.00^b^	0.29 ± 0.00^fg^
Hexanol	64.07 ± 0.02^f^	117.51 ± 1.12^c^	6.97 ± 0.04^j^	52.44 ± 0.07^g^	83.44 ± 0.14^d^	149.21 ± 0.07^a^	11.10 ± 0.04^j^	18.80 ± 0.04^i^	77.36 ± 0.05^e^	119.85 ± 2.89^c^	7.27 ± 0.06^j^	23.78 ± 0.05^h^	77.48 ± 0.04^e^	139.09 ± 0.07^b^
*Trans*−3‐hexenol	1.30 ± 0.00^c^	0.93 ± 0.02^e^	0.11 ± 0.00^h^	2.16 ± 0.00^a^	1.41 ± 0.01^b^	0.89 ± 0.00^e^	0.76 ± 0.00^f^	0.42 ± 0.00^g^	1.09 ± 0.00^d^	1.44 ± 0.06^b^	0.15 ± 0.00^h^	0.51 ± 0.00^g^	1.52 ± 0.00^b^	0.71 ± 0.00^f^
*Cis*−3‐hexenol	36.39 ± 0.04^c^	30.29 ± 0.28^e^	8.53 ± 0.27^j^	111.54 ± 0.23^a^	72.30 ± 0.00^b^	17.51 ± 0.04^h^	31.20 ± 0.05^e^	35.36 ± 0.00^c^	16.28 ± 0.03^h^	24.47 ± 1.20^f^	11.34 ± 0.17^i^	33.42 ± 0.23^d^	17.82 ± 0.25^h^	22.43 ± 0.13^g^
*Trans*−2‐hexenol	57.62 ± 0.25^f^	68.72 ± 0.82^e^	2.65 ± 0.26^h^	13.32 ± 0.25^g^	87.93 ± 0.39^c^	89.47 ± 0.26^c^	4.20 ± 0.04^h^	3.64 ± 0.02^h^	90.36 ± 0.21^c^	185.94 ± 2.15^b^	4.58 ± 0.23^h^	12.38 ± 0.30^g^	197.62 ± 0.28^a^	79.54 ± 0.27^d^
Acetic acid	0.14 ± 0.01^hi^	0.87 ± 0.02^c^	0.23 ± 0.01^ghi^	0.23 ± 0.02^ghi^	0.72 ± 0.02 cd	0.47 ± 0.02^ef^	0.26 ± 0.02^gh^	0.05 ± 0.00^i^	1.16 ± 0.03^b^	1.38 ± 0.12^a^	0.28 ± 0.01^fgh^	0.37 ± 0.02^fg^	0.75 ± 0.02 cd	0.63 ± 0.02^de^
Butyric acid	0.13 ± 0.00 ^cd^	0.39 ± 0.04^a^	0.00 ± 0.00^f^	0.15 ± 0.00^bc^	0.13 ± 0.01 cd	0.00 ± 0.00^f^	0.00 ± 0.00^f^	0.03 ± 0.02^ef^	0.21 ± 0.00^b^	0.00 ± 0.00^f^	0.07 ± 0.00^de^	0.10 ± 0.00 cd	0.00 ± 0.00^f^	0.00 ± 0.00^f^

Note

Different letters in the same line means significant difference between samples.

As shown in Table 4, the chemical composition of the volatile fraction of studied olive oil samples was variable, depending on both extraction system and cultivar. Regarding the major volatiles, their concentration varied significantly according to the extraction system. In fact, oils obtained by three‐phase centrifugation were the richest in *cis*‐3‐hexenol for the majority of cultivars and in hexanol for all cultivars, which emphasizes the perception of green, fruity, astringent, bitter, and pungent (Ben Lawlor et al., 2001; Brkić Bubola et al., 2012). Meanwhile oils produced by pressure process were richer in *trans*‐2‐hexenal for the majority of cultivars.

### 3.2 Influence of the cultivar and extraction process on the sensory attributes

The oils extracted from Chetoui and Arbequina cultivars by continuous processing system had a different aroma profile in comparison to those extracted by press system from Chemlali, Chetoui, and Leguim cultivars (Table 1).

The study of the effect of extraction system on our olive oils sensorial profile revealed a significant difference between oils obtained by three‐phase system and those extracted by press system. Oils obtained by centrifugation system were characterized by higher sensory scores for bitterness, fruitiness, and pungency than those obtained by press, except concerning Zalmati, Chemchali, and Arbequina VOOs. This is consistent with our previous work (Ben‐Hassine et al., 2013), where the processing system influenced the positive attributes (fruity, bitter, and pungent). In fact, the highest level of such attributes was observed in VOOs obtained with three‐phase centrifugation compared to two‐phase and super press (Ben‐Hassine et al., 2014). Meanwhile, it was reported in the literature that the oils extracted by the two‐phase centrifugal system exhibit a higher sensory score than after extraction by three‐phase due to the little amount of water added to the olive paste (Segura‐carretero et al., 2010). Regarding bitterness attribute, it was reported that its intensity was higher in two‐phase than in three‐phase decanter extracted oils (Clodoveo, 2012). In our study, the ANOVA test showed that the sensory profile of the present VOOs was more influenced by the extraction processing rather than the cultivar.

The bitterness and pungency perceived by taste are positive attributes for a VOO. These two sensory characteristics are closely connected by the qualitative–quantitative phenolic profile of the product (Reboredo‐Rodríguez et al., 2017). The latter present an important technological value due to their influence on sensory characteristics as well as the shelf life of virgin olive oil (Bendini et al., 2012). In addition, they are known for their health benefits and high antioxidant activities (Guerfel et al., 2009). VOO with a high intensity of bitterness, astringency, or pungency are hardly marketable in emergent markets. Consequently, blends should be made between such oils and nonbitter VOO. It is worthy to notice that the sensory attributes of EVOO are mainly correlated to the content of minor components such as phenolic and volatile compounds (Dabbou et al., 2010).

### 3.3 Principal component analysis (PCA)

**FIGURE 1:**
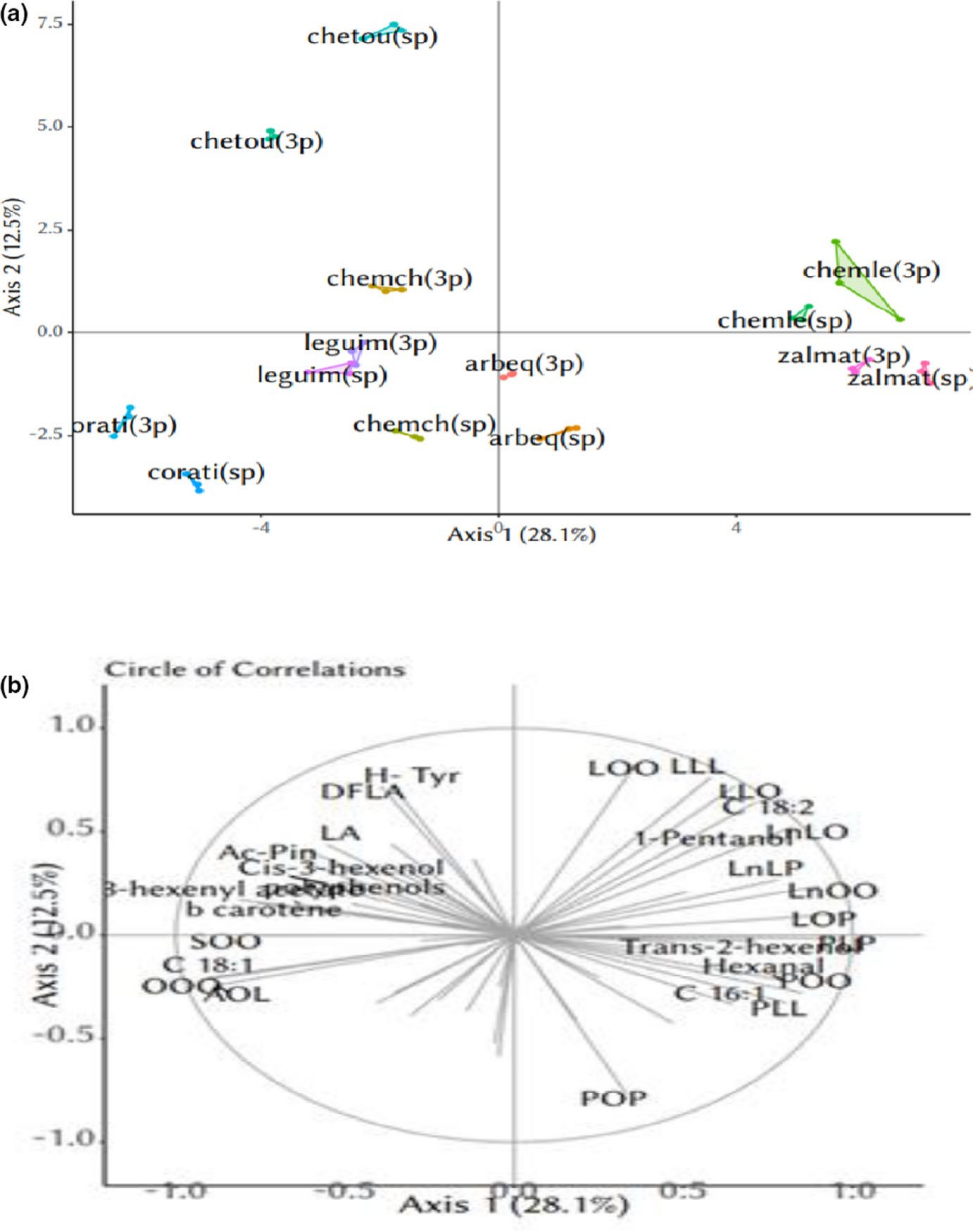
Principal component analysis based on physicochemical and sensorial attributes clustered by the AHC, (a) circle of correlations, (b) projection of olive oil samples on PC1 and PC2

According to the Principal component analysis (PCA) (Figure 1a), four main groups could be distinguished. Group 1 was formed by Chemlali 3p, Chemlali sp, Zalmati 3p, and Zalmati sp. Group 2 was composed of samples from Coratina cultivar (sp and 3p). Group 3 grouped the oils from Chetoui cultivar (sp and 3p) and group 4 was composed of Chemchali (sp and 3p), Leguim (sp and 3p), and Arbequina (sp and 3p) olive oils. Groups 1 and 2 were clearly separated according to PC1, while PC2 could separate Chetoui cultivar olive oils from the rest of olive oil samples.

Examining the correlation circle of variables under study (Figure 1b), we can deduce that among analyzed samples, group 1 olive oils are the richest in terms of hexanal, *trans*‐2‐hexenol, 1‐pentanol, TAG (LnLo, LOO, LLO, LLL, LnLP, LnOO, LOP, SOO), and C16:1. While group 2 oils were the richest in total polyphenols, Ac‐pin, LA, major TAGs (OOO, AOL, SOO), β‐carotene, C18:1 (marker of freshness), and volatile compound (*cis*‐3‐hexenol, 3‐hexenyl acetate). Regarding Chetoui olive oils presenting group 3, they were richer in H‐Tyr, DFLA, LOOO, and LLL. Samples of group 4 could not be well separated within PC1 and PC2.

### 3.4 Preference mapping

**FIGURE 2:**
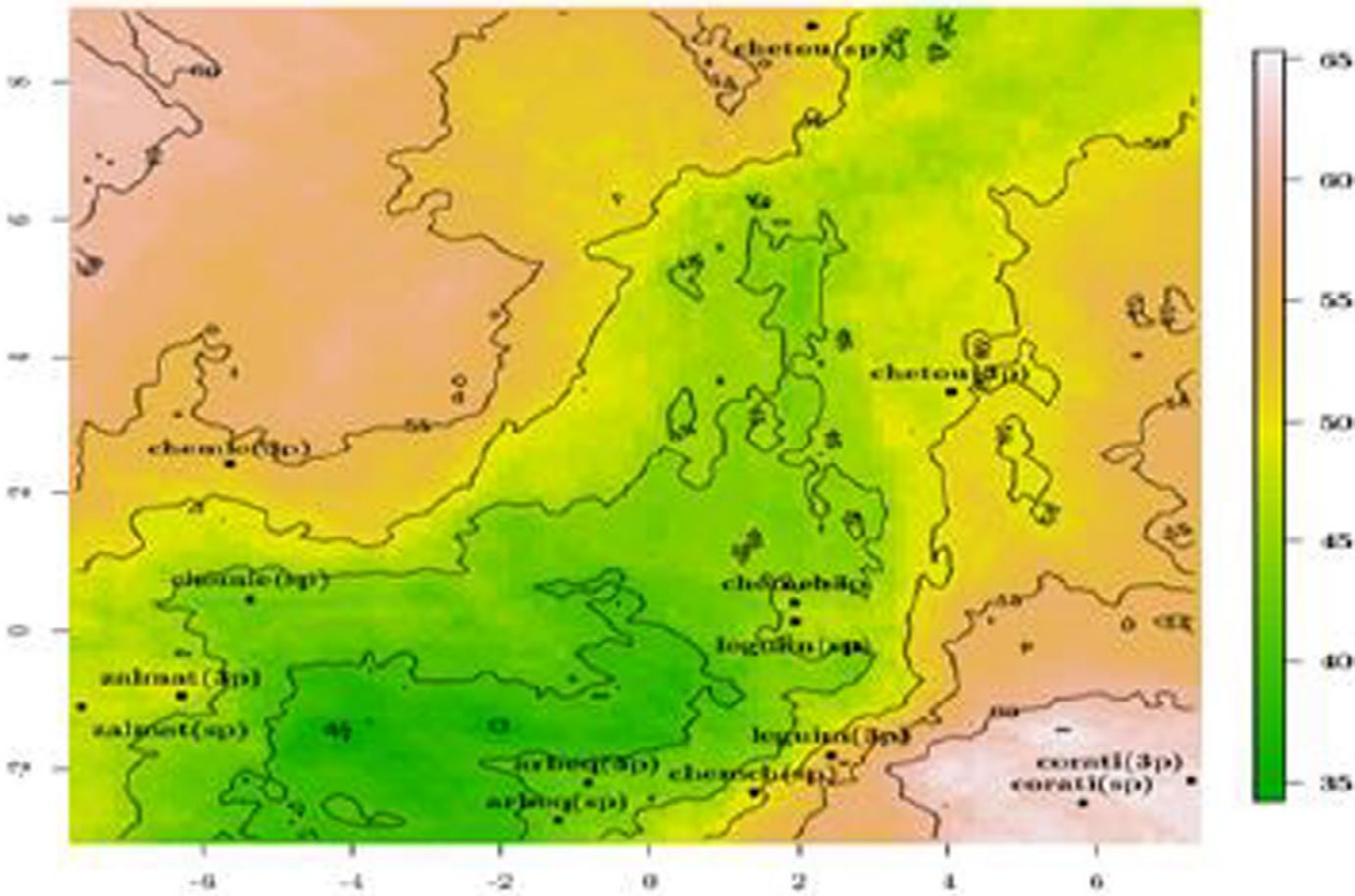
Preference mapping based on principal component analysis

To explore the consumer preferences toward the VOOs under study according to processing system and cultivar, a preference mapping approach based on PCA was used. External preference mapping is a very useful statistical technique which covers and measures the positive and negative drivers of olive oil sensory and chemical quality as perceived by consumer. On the map, the distance between each VOO product and the optimum (70%) was shown (Figure 2). Five main groups of VOOs could be observed on the map with a good segmentation according to the consumer preference score and chemical parameters. The first group is composed by the foreign olive oil Coratina (3p and sp) which was the most preferred one among the tested samples with 65% of consumer preference being the nearest to the optimum product preference (70%). The second group is also highly appreciated by consumers and presented the Tunisian olive oils Chemlali 3p, Leguim 3p, and Chetoui sp that attracted 50%–55% of the consumer appreciation (Figure 2). The third group composed of Chetoui 3p, Chemchali (sp and 3p), Zalmati 3p, Zalmati sp, and Leguim sp olive oils that attracted 45%–50% of consumer appreciation. The least appreciated group and the farthest from the optimum product is composed of Arbequina (sp and 3p) and Chemlali sp and was appreciated by 40%–45% of consumer choice. It is noteworthy to say that consumers could not distinguish between the two extractions systems (3p and sp).

Based on the PCA, the positive drivers of consumer choice for Coratina olive oil cultivar were its richness in total polyphenols (markers of bitterness and pungency), Ac‐pin, LA, major TAGs (OOO, AOL, SOO), β‐carotene, C18:1 (marker of freshness), and volatile compound *cis*‐3‐hexenol, 3‐hexenyl acetate, markers of green fruity, green leaves, green apple, cut grassy almond, and bitter perception.

The positive drivers for the olive oil Chemlali (3p) (52% of consumer appreciation) known by a profile of almond fruity green and low bitterness and pungency were its richness in hexanal related to the sensory perception of green fruity, green leaves, floral bitter, cut grassy, ripe apple, *trans*‐2‐hexenol, 1‐pentanol, TAG (LnLo, LOO, LLO, LLL, LnLP, LnOO, LOP, SOO), and C16:1. The Chetoui cultivar attracted consumers for its richness in H‐Tyr, DFLA (related to the sensory perception of astringency, bitterness, and pungency), and in LOOO and LLL.

The consumer choice for VOOs Zalmati (3p) and Zalmati (sp) is explained by the positive drivers that are higher amounts of TAG (LnLo, LOO, LLO, LLL, LnLP, LnOO, LOP, SOO) and C16:1 and the volatile compounds (1‐pentanol, *trans*‐2‐hexenol, hexanal) that explain the sensory perception of green fruity, sweet, green leaves, almond, and bitter as reported by (Angerosa 2002) and the lower amount of LA, Ac‐pin (bitter, pungent), *cis*‐3‐hexenol, 3‐hexenyl‐acetate (green fruity, green leaves, almond, bitter), low β‐carotene, and low amounts of OOO AOL C18:1. These latter explain Zalmati 3p and sp profiles: green fruity, low bitterness, pungency, which is not influenced by the processing system. However, the olive oil Chetoui 3p which showed 55% of consumer appreciation is characterized by the parameters negatively correlated with PC2.

The group composed of Chemchali 3p and Leguim sp olive oils attract 45% of consumer appreciation when its profile is characterized by higher amounts of TAG (OOO, AOL), β‐carotene, C18:1, and phenolic and volatile compounds (Ac‐pin, H‐tyr, LA, *cis*‐3‐hexenol, *cis*‐3‐hexenyl acetate), which denote the sensory perception of pungency, bitterness, fruity, apple, cut grassy, green leaves, almond, and the low amount of LOO, LLL, LLO, LnLP, LnOO, LOP, POO, PLL, C16:1, C18:2, and volatiles (1‐pentanol, *trans*‐2‐hexenol, hexanal) related to the green fruity and the bitter attributes. As reported by Angerosa et al. (2004), the latter compounds are the positive drivers of consumer appreciation which describe a sensory profile of green fruity, bitter, and pungent for Chemchali 3p and Leguim sp VOOs.

This study allowed us to distinguish that the foreign introduced variety Arbequina produced by sp or 3p is not in the tradition for Tunisian consumers. Regarding Chemchali sp VOO, the nonappreciation can be explained by the fact that this is a minor variety of olive oil as it represents only 2% of olive oil Tunisian production. It is not available in the market as a monovariable olive oil; it is mainly consumed in blends.

Preference mapping has been used extensively to describe the characteristics that contribute to consumer's acceptance or rejection by the identification of both positive and negative drivers of liking (Delgado & Guinard, 2011). Concerning the sensorial attributes, researches revealed that the most valued attributes from the consumer viewpoints are the “ripe fruity” and “sweet” (Valli et al., 2014), high intensity for color, odor, taste, and flavor, and pungent and floral series (Zamuz et al., 2020). However, the bitter (Zamuz et al., 2020) and pungent positive sensorial attributes are rejected by the consumer (Delgado & Guinard, 2011). This rejection could be related to the unfamiliarity of consumers (Valli et al., 2014) with these attributes and their lack of knowledge concerning the relation among bitter and pungent attributes and nutritional and healthy properties of olive oils (Zamuz et al., 2020). In Tunisia, researches conducted by Mtimet et al. (2013) concluded that Tunisian consumers have a good knowledge of the characteristics of olive oil.

## 4 CONCLUSION

The consumer preference evaluation for the studied Tunisian and foreign VOOs showed that liking was related to chemical and sensory profiles and varied among consumers as revealed by the preference mapping. The study identifies the key liking drivers, such as polyphenols, oleic acid, and good aroma compounds namely *cis*‐3‐hexenol and 3‐hexenyl acetate. Among studied samples, Coratina VOOs were the most appreciated followed by Chemlali. In general, consumers appreciated the fruity attribute and, in part, the pungent sensation, whereas they recognize bitterness as a negative attribute. Processing systems are found to be the main drivers of consumers' choice in favor of the discontinuous system (sp). We believe that these findings ought to have been supplemented with an evaluation of foreign consumers' preference in order to accommodate their need according to their chemical and sensory olive oil profile, since Tunisian olive oils are widely exported around the world. Furthermore, it could be useful if we explore preference evaluation according to consumer characteristics (gender, age, etc.). This remains a subject for future investigation.

## CONFLICT OF INTEREST

The authors declare that they have no conflict of interest.

## Acknowledgements

Required.

## Data Availability

Data are available on request by the first author Dr. Kaouther Ben‐Hassine (kaoutheragro@yahoo.fr).
